# High G9a Expression in DLBCL and Its Inhibition by Niclosamide to Induce Autophagy as a Therapeutic Approach

**DOI:** 10.3390/cancers15164150

**Published:** 2023-08-17

**Authors:** Chin-Mu Hsu, Kung-Chao Chang, Tzer-Ming Chuang, Man-Ling Chu, Pei-Wen Lin, Hsiao-Sheng Liu, Shih-Yu Kao, Yi-Chang Liu, Chien-Tzu Huang, Min-Hong Wang, Tsung-Jang Yeh, Yuh-Ching Gau, Jeng-Shiun Du, Hui-Ching Wang, Shih-Feng Cho, Chi-En Hsiao, Yuhsin Tsai, Samuel Yien Hsiao, Li-Chuan Hung, Chia-Hung Yen, Hui-Hua Hsiao

**Affiliations:** 1 Division of Hematology and Oncology, Department of Internal Medicine, Kaohsiung Medical University Hospital, Kaohsiung 807, Taiwan; e12013@gmail.com (C.-M.H.); benjer6@gmail.com (T.-M.C.); ycliu@cc.kmu.edu.tw (Y.-C.L.); dhlsy01128@gmail.com (M.-H.W.); aw7719@gmail.com (T.-J.Y.); cheesecaketwin@gmail.com (Y.-C.G.); ashiun@gmail.com (J.-S.D.); joellewang66@gmail.com (H.-C.W.); sifong96@gmail.com (S.-F.C.); 2 Department of Pathology, Kaohsiung Medical University Hospital, Kaohsiung 807, Taiwan; changkc@mail.ncku.edu.tw; 3 Department of Pathology, College of Medicine, Kaohsiung Medical University, Kaohsiung 807, Taiwan; 4 Department of Pathology, National Cheng Kung University Hospital, College of Medicine, National Cheng Kung University, Tainan 704, Taiwan; 5 M.Sc. Program in Tropical Medicine, College of Medicine, Kaohsiung Medical University, Kaohsiung 807, Taiwan; c_manling@yahoo.com.tw (M.-L.C.); peiwen349@gmail.com (P.-W.L.); hsliu713@kmu.edu.tw (H.-S.L.); 6 Center for Cancer Research, Kaohsiung Medical University, Kaohsiung 807, Taiwan; chyen@kmu.edu.tw; 7 Cancer Center, Kaohsiung Medical University Hospital, Kaohsiung 807, Taiwan; scyy3266@gmail.com; 8 Faculty of Medicine, College of Medicine, Kaohsiung Medical University, Kaohsiung 807, Taiwan; 9 Graduate Institute of Clinical Medicine, College of Medicine, Kaohsiung Medical University, Kaohsiung 807, Taiwan; 10 Department of Molecular and Cell Biology, University of California, Berkeley, CA 94720, USA; hsiaopaulabear@gmail.com; 11 Graduate Institute of Chinese Medicine, School of Chinese Medicine, China Medical University, Taichung 404, Taiwan; yhtsai@mail.cmu.edu.tw; 12 Department of Biology, University of Rutgers-Camden, Camden, NJ 08102, USA; ucdsacnyu@gmail.com; 13 Long-Term Care and Health Management Department, Cheng Shiu University, Kaohsiung 833, Taiwan; 0649@gcloud.csu.edu.tw; 14 Graduate Institute of Natural Products, College of Pharmacy, Kaohsiung Medical University, Kaohsiung 807, Taiwan

**Keywords:** DLBCL, G9a/EHMT2, niclosamide, autophagy, immunohistochemistry, TissueFAXS PLUS, HistoQuest

## Abstract

**Simple Summary:**

Diffuse large B-cell lymphoma (DLBCL) is a prevalent hematological malignancy which is characterized by rapid cancer cell growth and aggressive progression. The standard treatment is R-CHOP, which offers limited prognosis improvement for only 60% of patients. This study aimed to identify DLBCL tumor markers and to explore cytotoxic medicine. The results demonstrated significant upregulation of G9a/EHMT2 mRNA in DLBCL. Niclosamide effectively suppressed G9a expression, modulated autophagy-related gene expression (p62, BECN1, and LC3), and impeded DLBCL cell proliferation. Analysis of clinical specimens and patients’ information revealed a positive correlation between G9a protein levels and DLBCL staging, indicating its potential as a prognostic biomarker. Our study provides valuable insight that highlights the potential of G9a as a therapeutic target and niclosamide as a potential treatment to combat DLBCL.

**Abstract:**

Background: Diffuse large B-cell lymphoma (DLBCL) is a malignant lymphoid tumor disease that is characterized by heterogeneity, but current treatment does not benefit all patients, which highlights the need to identify oncogenic genes and appropriate drugs. G9a is a histone methyltransferase that catalyzes histone H3 lysine 9 (H3K9) methylation to regulate gene function and expression in various cancers. Methods: TCGA and GTEx data were analyzed using the GEPIA2 platform. Cell viability under drug treatment was assessed using Alamar Blue reagent; the interaction between G9a and niclosamide was assessed using molecular docking analysis; mRNA and protein expression were quantified in DLBCL cell lines. Finally, G9a expression was quantified in 39 DLBCL patient samples. Results: The TCGA database analysis revealed higher G9a mRNA expression in DLBCL compared to normal tissues. Niclosamide inhibited DLBCL cell line proliferation in a time- and dose-dependent manner, reducing G9a expression and increasing p62, BECN1, and LC3 gene expression by autophagy pathway regulation. There was a correlation between G9a expression in DLBCL samples and clinical data, showing that advanced cancer stages exhibited a higher proportion of G9a-expressing cells. Conclusion: G9a overexpression is associated with tumor progression in DLBCL. Niclosamide effectively inhibits DLBCL growth by reducing G9a expression via the cellular autophagy pathway; therefore, G9a is a potential molecular target for the development of therapeutic strategies for DLBCL.

## 1. Introduction

Diffuse large B-cell lymphoma (DLBCL) is an aggressive malignant lymphoid neoplasm, which accounts for 40% of all malignant lymphoid neoplasms and includes morphologically and molecularly heterogeneous disease subtypes [1,2]. According to gene expression profiling, different forms of DLBCL are subdivided into germinal center B-cell-like (GCB-DLBCL) and activated B-cell-like (ABC-DLBCL) subtypes with distinct genetic aberrations [3]. The GCB-DLBCL subtype exhibits a better prognosis than the ABC subtype, and recently, genetic aberrations have been integrated into the cell of origin (COO) classification [4,5,6]. Over 30% of DLBCL patients experience recurrent or progressive disease after R-CHOP treatment [4,7,8,9]. Therefore, there is a need for novel therapeutic strategies that target signaling pathways to improve DLBCL therapies.

Genomic aberrations involving epigenetic modifier genes in DLBCL have been associated with different clinical outcomes in specific epigenetic subgroups [10,11]. In the epigenetic modifier genes, G9a, also known as EHMT2 or KMT1C, is a histone methyltransferase that forms part of the polycomb repressive complex-2 (PRC2) involved in regulating gene expression by catalyzing the transfer of the methyl group from s-adenosylmethionine (SAM) to the lysine 9 and/or lysine 27 on histone 3 (H3K9 and/or H3K27), leading to mono-, di- or trimethylation histone states [12,13]. Recently, G9a has been found to be overexpressed in malignant cells and to play an important role in tumor progression and metastasis, particularly, liver, renal, lung, and gastric cancers [14,15,16,17,18]. G9a acts at various levels to promote cancer progression through molecular mechanisms such as enhancing stemness, epithelial–mesenchymal transition, and drug resistance [19,20,21]. G9a inhibition can reduce cancer cell proliferation and can delay tumor metastasis, suggesting that G9a may be a potential target for cancer treatment [22,23]. However, the development of G9a inhibitors is challenging.

Niclosamide is a parasitic drug that was approved by the United States Food and Drug Administration (the US FDA) in 1982 for the treatment of tapeworm infection [24]. It has also been used in multiple target therapies for the inhibition of bacterial and viral infections, metabolism regulation, and as an anticancer drug [25,26,27,28,29]. In malignant tumors, niclosamide inhibits Wnt/β-catenin, STAT3, mTOR, NF-κB, and the BCL2 family to induce tumor cell death in adrenocortical carcinoma, lung cancer cells, leukemia, hepatocellular carcinoma, and breast cancer [25,26,27,30,31]. It also acts synergistically in combination with other chemotherapeutic drugs such as cisplatin and erlotinib to regulate mitochondrial dysfunction [31,32,33].

Based on our understanding, G9a has been closely implicated in tumorigenesis and cancer prognosis. Concurrently, niclosamide has demonstrated inhibitory effects on cancer cell proliferation. However, to date, the association between G9a and niclosamide has not been investigated; therefore, this study aims to understand their relationship when utilized as therapeutic treatment. In this study, G9a expression and activity were investigated in human DLBCL disease. Furthermore, we studied the effects of niclosamide on DLBCL and G9a and their associated signaling pathways. Niclosamide reduced G9a protein levels in a dose-dependent manner without affecting mRNA levels, indicating that niclosamide affects G9a protein stability rather than transcription. Additionally, niclosamide increased LC3I/II, a marker of autophagy induction. Autophagy is a cellular process that degrades damaged or unwanted organelles and proteins through lysosomal degradation. It can exert both pro-survival and pro-death roles in cancer cells depending on the context and stimuli. We hypothesize that niclosamide induces autophagy in DLBCL cells as a response to G9a inhibition, which may lead to cell death or senescence. Niclosamide demonstrates the ability to (1) inhibit G9a expression, (2) modulate autophagy-related genes, and (3) suppress the growth of DLBCL cells, signifying a novel therapeutic direction for DLBCL treatment by targeting G9a modulation and highlighting the potential of niclosamide as a pharmacological intervention for DLBCL. It provides a new avenue for further research and clinical exploration. Leveraging the repurposing potential of niclosamide, it has the opportunity to expand the efficacy of current treatment strategies and its application can be extended to other cancer diseases via G9a regulation.

## 2. Materials and Methods

### 2.1. GEPIA Analysis

The GEPIA2 (Gene Expression Profiling Interactive Analysis; http://gepia2.cancer-pku.cn/#index (accessed on 23 May 2023)) online software was utilized to analyze RNA-seq data and to investigate the relationship between EHMT2/G9a and the expression of tumor markers in TGGA and GTEx datasets [34]. Additionally, a boxplot was generated using GEPIA2 to compare gene expression in all cancer types with genes having |log2FC| values >1 and q-values < 0.01 being considered differentially expressed. The Spearman method was chosen for the correlation coefficient analysis between EHMT2/G9a and the expression of specific markers. These databases provide customizable functions, including tumor/normal differential expression analysis and patient survival analysis, which aids in the analysis of RNA-seq data.

### 2.2. Cell Culture

Human DLBCL cell lines (U2932, HT, SU-DHL-5, and RC-K8) were purchased from the American Type Culture Collection (ATCC, Rockville, MD, USA) and the Bioresource Collection and Research Center (BCRC, Hsinchu, Taiwan). The DLBCL cell lines were cultured at 37 °C in a humidified 5% CO_2_ incubator in RPMI 1640 (HCC38) supplemented with 10% fetal bovine serum ((FBS) Merck, Darmstadt, Germany), 2% penicillin-streptomycin ((P/S) Gibco Thermo Fisher Scientific, Waltham, MA, USA), 10 mM HEPES (Gibco Thermo Fisher Scientific), 2 mM L-glutamine (Gibco Thermo Fisher Scientific), 2 g/L D-glucose (Sigma Aldrich, St Louis, MO, USA), and 1 mM Sodium Pyruvate (Gibco Termo Fisher Scientific).

### 2.3. Cell Viability

Human U2932, HT, SU-DHL-5, and RC-K8 DLBCL cells were seeded into 96-well microtiter plates at a density of 5000 cells per well and incubated in RPMI-1640 medium with 10% FBS in a final volume of 100 μL. Subsequently, the cells were treated with increasing concentrations of niclosamide or DMSO for 72 h. Cell viability was quantified using an Alamar Blue viability assay kit (DAL1025, Thermo Fisher Scientific, Waltham, MA, USA) according to the manufacturer’s protocol. The optical density was measured at 570 nm or 590 nm using a microplate reader, and cell viability was expressed as a percentage of the control cells and the drug concentration. The experiment was conducted in triplicate.

### 2.4. Immunoblotting

Protein expression was investigated by immunoblotting. DLBCL cells were treated with niclosamide for 24 h, then washed twice with ice-cold PBS. Proteins were isolated using RIPA lysis buffer (50 mM Tris-HCl, pH 7.5, 150 mM NaCl, 0.5% sodium deoxycholate, 1% NP-40, and 0.1% SDS) (Thermo Fisher Scientific, Waltham, MA, USA) containing a protease inhibitor cocktail (Cyrusbio, New Taipei, Taiwan). The protein concentration was determined by the Bradford assay (Bio-Rad, Contra Costa County, CA, USA), and the proteins were separated by 10% SDS-PAGE before immunoblotting using the following antibodies: G9a (GeneTex, Hsinchu, Taiwan) and LC3I/II (Cell Signaling Technology, Topsfield, MA, USA). GAPDH (Cell Signaling Technology, Topsfield, MA, USA) was used as a loading control. The protein bands were quantified using the ImageJ image analysis software (version 1.53k, National Institutes of Health, Bethesda, MD, USA), and the experiment was performed in triplicate.

### 2.5. RT−qPCR

Total RNA was extracted from the DLBCL cells using TOOLSmart RNA Extractor reagent (TOOLS, BIOTOOLS, New Taipei, Taiwan) and 1 μg of RNA was reverse transcribed using a High-Capacity cDNA Reverse Transcription cDNA Synthesis Kit (Applied Biosystem, Foster City, CA, USA) according to the manufacturer’s protocol. RT-qPCR was performed on a QuantStudio real-time PCR system (Applied Biosystem, Foster City, CA, USA) using Eztime Real-Time PCR Premix (2 × SYBR Green reagent, Yeastern Biotech, New Taipei, Taiwan) with the primers described in Table 1. The cycling conditions included 95 °C for 10 min for pre-incubation; 40 cycles of 95 °C for 10 s, 57 °C for 10 s, and 72 °C for 10 s; 65 °C for 10 s for melting curves; and 40 °C for 30 s for cooling. Relative RNA expression levels were calculated from Ct values according to the ΔCt method and normalized to control GAPDH RNA levels.

### 2.6. Collection of Clinical Samples

A total of 39 consecutive DLBCL patients confirmed by lymph node pathological examination at the Kaohsiung Medical University Chung-Ho Memorial Hospital between July 2015 and October 2020 were enrolled in this study. Tumor staging was evaluated according to the seventh and eighth editions of the American Joint Committee on Cancer/International Union for Cancer Control (AJCC/UICC) TNM staging system. This retrospective study was approved by the Institutional Review Board of Kaohsiung Medical University Chung-Ho Memorial Hospital (KMUHIRB-E(I)-20190367 and KMUHIRB-E(I)-20210119).

### 2.7. Immunohistochemistry

Immunohistochemistry (IHC) was performed on 4 μm thick, formalin-fixed paraffin-embedded (FFPE) sections using monoclonal mouse anti-G9aEu-HMTase 2 antibody (1:100 dilution) (R&D Systems, Cat. #PP-A8620A-00), the Bond Polymer Refine Detection Kit (Leica Biosystems Newcastle Ltd., Newcastle upon Tyne, UK) and a BOND-MAX Automated IHC Staining System (Leica Biosystems Newcastle Ltd., Melbourne, Australia) according to the following protocol. Tissues were deparaffinized with xylene and pretreated with epitope retrieval solution 1 (ER1) (citrate, pH 6.0, 100 °C, 20 min) followed by incubation with the primary antibody at room temperature for 30 min. Subsequently, tissues incubated with polymer for 15 min and hydroperoxide blocking for 5 min before developing with 3,3′-diaminobenzidine chromogen (DAB) for 10 min. Counterstaining was performed with hematoxylin for 5 min. Squamous cell carcinoma tissues were used as positive and negative controls. All histological and immunohistochemical slides were reviewed independently by the pathologists.

### 2.8. Image Analysis Using the TissueFAXS PLUS System

The slides were digitally scanned using the TissueFAXS PLUS system (version 4.2.6245.1019, TissueGnostics, Vienna, Austria) on a Zeiss Observer microscope and the HistoQuest software (version 4.0.4.0158, TissueGnostics, Vienna, Austria) was used to analyze the G9a and hematoxylin staining and cell counts. Image analysis was performed in different regions of interest (ROI) in each image slide. G9a positive cell expression was calculated in percentages throughout the ROIs, and at least three representative areas were measured. The pixels were converted to grayscale and assigned an arbitrary number relating to the staining intensity, and then values were optimized by counting the number of DAB-positive and hematoxylin-positive events.

### 2.9. Molecular Docking

Molecular docking was conducted using the CB-DOCK2 server (https://cadd.labshare.cn/cb-dock2/php/index.php) based on AutoDock Vina [35]. The server performed blind docking of the ligand with the protein and identified potential binding cavities. The 3D structure of human G9a (PDB ID: 2O8J) was retrieved from the Protein Data Bank (https://www.rcsb.org/) [36]. The ligand (niclosamide) was obtained from PubChem [37]. G9a and niclosamide molecules were both uploaded to the CB-Dock2 server in .pdb file format, and the default setting selected five cavities for the docking study.

### 2.10. Statistical Analysis

The data were presented as mean ± standard deviation (mean ± SD). One-way ANOVA was used to analyze the differences between groups and a two-tailed unpaired Student’s *t*-test was used to analyze the difference between two groups (* *p* < 0.05, ** *p* < 0.01, and *** *p* < 0.001). All statistical analyses were performed using the SPSS software version 19.

## 3. Results

### 3.1. G9a Is Highly Expressed in DLBCL Patient Samples

**Figure 1:**
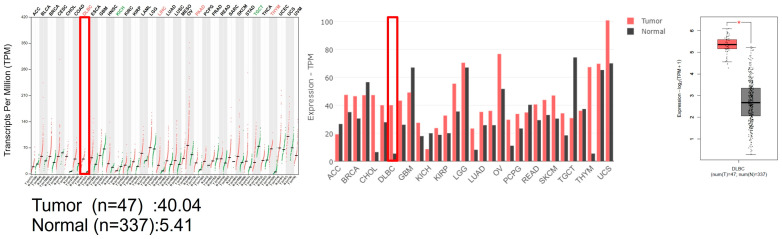
The gene expression profiling interactive analysis through the GEPIA software. DLBCL data were analyzed in TCGA and GTEx databases through the GEPIA web-based software, showing that G9a was more highly expressed in DLBCL tumor tissues than in normal tissues. Abbreviations: The Cancer Genome Atlas (TCGA), the Genotype-Tissue Expression (GTEx), adrenocortical carcinoma (ACC), bladder urothelial carcinoma (BLCA), breast invasive carcinoma (BRCA), cervical squamous cell carcinoma and endocervical adenocarcinoma (CESC), cholangiocarcinoma (CHOL), colon adenocarcinoma (COAD), lymphoid neoplasm diffuse large B-cell lymphoma (DLBC), esophageal carcinoma (ESCA), glioblastoma multiforme (GBM), head and neck squamous cell carcinoma (HNSC), kidney chromophobe (KICH), kidney renal clear cell carcinoma (KIRC), kidney renal papillary cell carcinoma (KIRP), acute myeloid leukemia (LAML), brain lower grade glioma (LGG), liver hepatocellular carcinoma (LIHC), Lung adenocarcinoma (LUAD), lung squamous cell carcinoma (LUSC), mesothelioma (MESO), ovarian serous cystadenocarcinoma (OV), pancreatic adenocarcinoma (PAAD), pheochromocytoma and paraganglioma (PCPG), prostate adenocarcinoma (PRAD), rectum adenocarcinoma (READ), sarcoma (SARC), skin cutaneous melanoma (SKCM), stomach adenocarcinoma (STAD), testicular germ cell tumors (TGCT), thyroid carcinoma (THCA), thymoma (THYM), uterine corpus endometrial carcinoma (UCEC), uterine carcinosarcoma (UCS), uveal melanoma (UVM) (*, *p* < 0.05).

The GEPIA2 software provides interactive analysis of gene expression data from the Cancer Genome Atlas (TCGA) and the Genotype-Tissue Expression (GTEx) databases. Among the genes that showed differential expression in DLBCL tumor versus normal tissues, we focused on EHMT2/G9a, a histone methyltransferase that regulates chromatin structure and gene transcription. G9a was more highly expressed in DLBCL tumor tissues (*n* = 47) than in normal tissues (*n* = 337), with RNA transcripts per million (TPM) of 40.04 and 5.41, respectively (Figure 1). G9a was significantly upregulated in DLBCL patients via GEPIA2 analysis, suggesting that EHMT2/G9a may play a role in DLBCL pathogenesis and could be a potential target for epigenetic therapy.

### 3.2. Niclosamide Inhibited DLBCL Cell Line Proliferation in a Time- and Concentration-Dependent Manner and Molecular Docking Study with G9a

**Figure 2:**
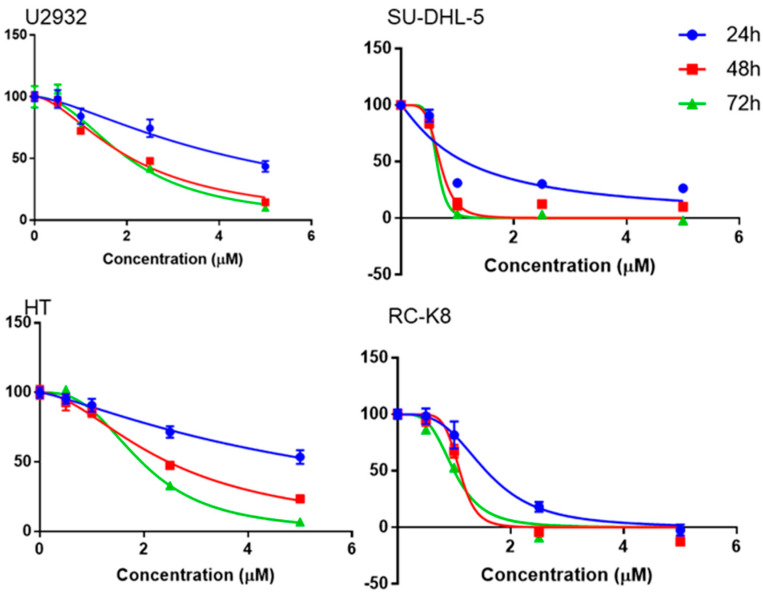
Cell viability assay of niclosamide-treated U2932, HT, SU−DHL−5, and RC−K8 cells over time and concentrations.

Niclosamide was used to explore the proliferation of DLBCL cells at various concentrations and time points, showing that niclosamide inhibited DLBCL cell proliferation in a concentration- and time-dependent manner (Figure 2). Regardless of the incubation time, niclosamide inhibited the growth of DLBCL cells at concentrations of 2.5 μM or above. Moreover, a 30% reduction in DLBCL cells could be achieved after 24 h of treatment with 2.5 μM of niclosamide; the inhibitory effect on DLBCL cells increased in a dose-dependent manner. To analyze and predict the potential interaction between G9a and niclosamide, we conducted a molecular docking study using the CB-DOCK2 web server. The 3D structure of G9a (PDB ID: 2O8J) was utilized for the analysis. The results indicated that the most probable binding pose of niclosamide exhibited a Vina score of −7.9. The binding interaction between niclosamide and G9a can be observed in Figure S3.

### 3.3. Niclosamide Treatment Reduced G9a and Induced LC3 Proteins

**Figure 3:**
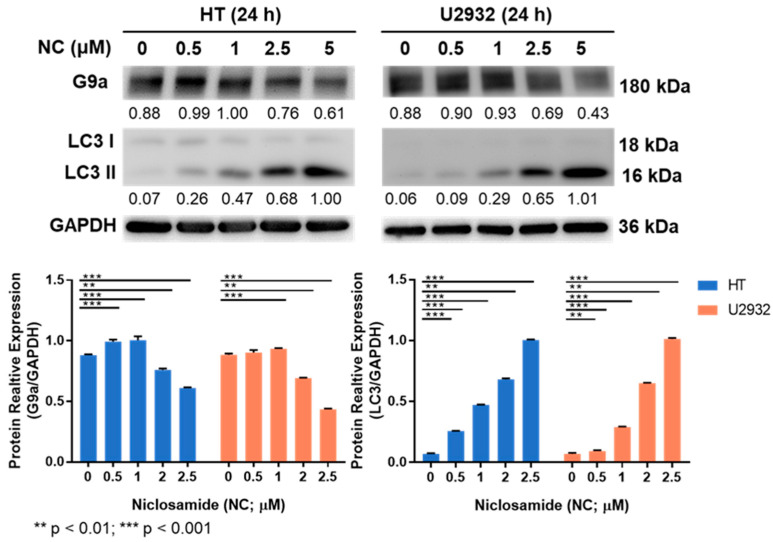
G9a expression was inhibited and LC3 genes were increased after niclosamide treatment.

G9a protein expression decreased as the niclosamide dose increased (Figure 3), suggesting that niclosamide interferes with the activity and stability of G9a, which may have implications for its anticancer mechanism of action. Furthermore, the expression of the autophagy signal gene, LC3I/II, increased with the increasing concentration of niclosamide compared to the untreated control (Figure 3).

### 3.4. Niclosamide Induced p62 and BECN1 Genes Expressed in DLBCL Cells

**Figure 4:**
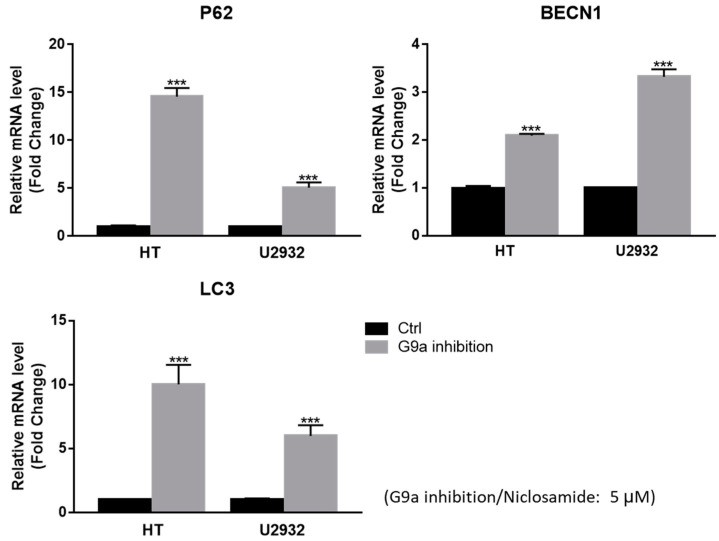
RT-qPCR showed that niclosamide can induce p62, BECN1, and LC3 expression in DLBCL cells (***, *p* < 0.001).

Numerous genes participate in the signaling pathways regulating autophagy, including the autophagy-associated genes p62 and BECN1. In our study, treatment with niclosamide at 5 μM induced the expression of p62 and BECN1 genes (*p* < 0.001), and this was accompanied by significant LC3 activation after 24 h of treatment, indicating that both HT and U2932 cells underwent autophagy (Figure 4). These results suggest that the regulation of both p62 and BECN1 genes is involved in the autophagy induced by niclosamide treatment in DLBCL cells.

### 3.5. G9a Expression Is Associated with Advanced-Stage DLBCL

**Figure 5:**
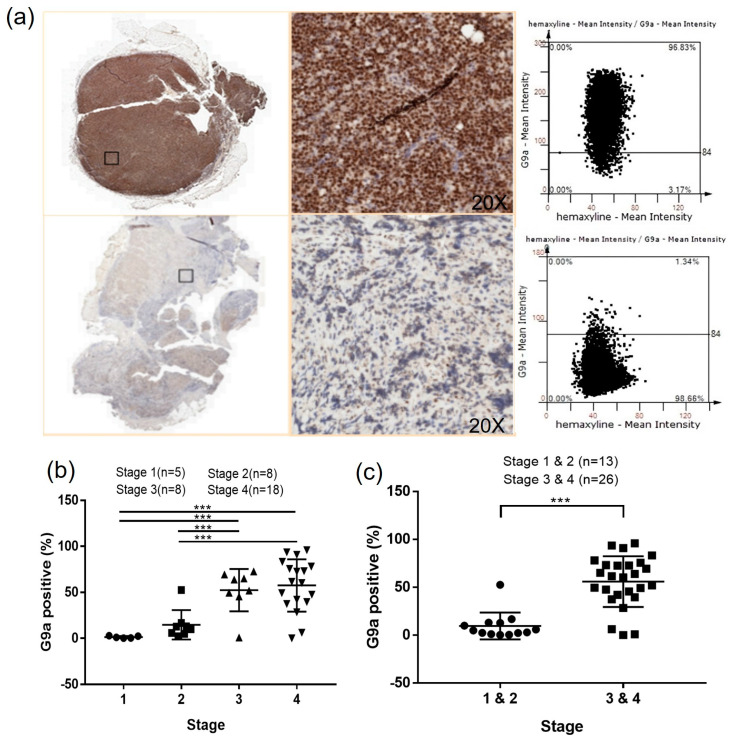
(**a**) Histological staining was scanned using the TissueFAXS PLUS system and analyzed by the HistoQuest software; (**b**) higher G9a expression in the advanced stage DLBCL; (**c**) G9a expression is higher in DLBCL stages III and IV than in stages I and II (***, *p* < 0.001).

The G9a protein expression in DLBCL patient samples was analyzed by IHC staining (Figure 5). The HistoQuest software was used to quantify the number of G9a-positive cells (Figure 5a) showing that higher G9a expression was accompanied by increased tumor malignancy in DLBCL patients (*p* < 0.001) (Figure 5b), with higher G9a expression in DLBCL stages III and IV compared to the stage I and II samples (*p* < 0.001) (Figure 5c).

### 3.6. The Clinical Characteristics Analysis Presents a Significant Difference in the Ann Arbor Stage

The two groups of patients with different G9a levels are compared in Table 2, showing that the group expressing G9a >average has a higher proportion of males compared to the G9a <average group (52.38% vs. 38.89%), but the difference is not statistically significant (*p* = 0.878). The average age of both groups was not significantly different (59.76 ± 18.86 vs. 61.22 ± 12.69, *p* = 0.776) and there was no significant difference in ECOG status (*p* = 0.425). However, there is a significant difference in the Ann Arbor stage (*p* = 0.00166), with the G9a >average group having more patients with stage III/IV disease and the G9a <average group having more patients with stage I/II disease. The G9a >average group also has significantly higher hemoglobin levels compared to the G9a <average group (12.91 ± 1.98 vs. 11.20 ± 1.73, *p* = 0.01), but there are no significant differences in BMI, white blood cell count, platelet count, LDH, or beta-2 microglobulin levels between the two groups.

## 4. Discussion

DLBCL is a non-Hodgkin lymphoma (NHL) characterized by invasive B cells and it occurs throughout Asia-Pacific and Western countries. In Taiwan, DLBCL patients account for 50–60% of NHL cases, with 80% of patients newly diagnosed between the ages of 50 and 80 years old [38,39,40]. In our collected DLBCL specimens, the average age and median age were both 60 years old, with patients aged 50 or above accounting for 74.36% of the total. Regarding molecular characteristics, DLBCL is mainly divided into two subtypes, i.e., activated B-cell-like (ABC) and germinal center B-cell-like (GCB), based on gene expression profiling (GEP) and gene activation status [41]. In terms of our subtype analysis, the ABC type plus non-GCB type accounted for 71.79% (*n* = 28/39) of the total, while the GCB type accounted for 28.21% (*n* = 11/39), indicating that among Taiwanese DLBCL patients, the majority were the ABC type, and these findings were consistent with previous results [39,42].

Histone methylation is a crucial regulatory mechanism in cancer progression. Several methyltransferases, including G9a, have been extensively investigated as potential therapeutic targets across various cancer tissues. Previous studies have demonstrated an association between G9a expression and patient survival outcomes in DLBCL. Higher expression levels of the G9a protein are correlated with a poorer prognosis in patients, as evidenced by worsened overall survival (OS) and progression-free survival (PFS) [43]. In our study, the data presented showed higher G9a expression in RNA profiling of DLBCL compared to normal control tissues, based on an analysis using the GEPIA software through the TCGA and GTEx databases. Additionally, it demonstrated that G9a was highly expressed in various cancerous tissues (Figure 1 and Figure S1). After utilizing immunohistochemistry to evaluate G9a expression in DLBCL tissue sections, we employed the HistoQuest software for a quantitative analysis and observed a statistically significant increase in the number of G9a-positive cells with cancer stage progression. Particularly, in stage III and IV cancers, there was a higher distribution of G9a-positive cancer cells within the cancer tissues (Figure 5b,c). We quantified the proportion of G9a-expressing cells relative to the total number of cells, which had an average value of 40 (Table 2). Based on this average value, patients were stratified into two groups and their clinical and biochemical data were compared, revealing that patients with G9a expression >40 exhibited a more advanced cancer stage compared to those with G9a expression <40, and there was a significant inter-group difference (*p* = 0.00166). Further analysis demonstrated that up to 95.24% of the patients in the G9a expression >40 group were classified as stage III or IV, suggesting a statistically significant correlation between G9a expression and advanced cancer staging in stage III and IV patients (*p* = 0.0158). When DLBCL was grouped based on two subtypes, i.e., GCB and ABC, the number of G9a-expressing cells was significantly higher in the ABC subtype compared to the GCB subtype (Figure S2). Generally, patients with abundant G9a expression tended to have a poorer prognosis. Furthermore, our data indicated that this prognostic difference may be associated with an increased number of cells expressing G9a, particularly in advanced cancer stages and the ABC subtype. Targeting the inhibition of G9a methyltransferase presents a potential avenue for cancer treatment, particularly within the context of DLBCL.

R-CHOP is the most common regimen and the standard treatment for DLBCL therapy but there are still 30–40% of cases that relapse or are refractory after therapy [40,44]. In recent years, numerous novel drugs and therapeutic modalities have been investigated in clinical trials for the management of DLBCL and lymphoma [45]. However, most clinical trials that are assessing histone methylation transferase inhibitors are currently in Phase I/II. The approval process for new drugs often entails rigorous and prolonged evaluation to ensure compliance with regulatory requirements. Moreover, the marketing of novel drugs necessitates a period of usage and surveillance to ascertain their effectiveness and safety [46]. It is noteworthy that the utilization of novel drugs may incur exorbitant costs that exceed the financial capabilities of ordinary households [45,47]. Drug repurposing is an emerging strategy that involves identifying new therapeutic uses for approved or investigational drugs [48,49]. Compared to the traditional drug development process which is characterized by high costs, prolonged timelines, and high failure rates, drug repurposing offers several advantages. By leveraging existing knowledge about a drug’s safety and pharmacology, repurposing can streamline the development process, thus reducing research and development costs. In addition, repurposing can enhance patient access to effective treatments as an important approach for addressing unmet medical needs and optimizing healthcare outcomes.

Niclosamide (also known as BAY2353, Niclocide, NSC 178296, CAS No. 50-65-7) is an antihelminthic agent for the treatment of tapeworm infections, which is very safe and has low toxicity to humans; it was approved by the United States Food and Drug Administration (US FDA) in 1982 [50]. Niclosamide can inhibit DNA replication and STAT3 phosphorylation to alter metabolism in type 2 diabetes (T2D) mice [51]. In addition, niclosamide has exhibited significant anticancer activity against diverse cancer types [26,27,30,52,53,54,55,56]. In hepatocellular carcinoma cells, niclosamide inhibits cell viability, clone formation, and induces apoptosis by deactivating STAT3 phosphorylation and downregulating antiapoptotic proteins, Mcl-1, and survivin [27]. Additionally, in adrenocortical carcinoma, niclosamide suppresses cellular proliferation by arresting the cell cycle in the G1 phase, inducing caspase-dependent apoptosis, and reducing β-catenin levels, thereby mitigating cellular migration and epithelial-to-mesenchymal transition [30]. In leukemia, niclosamide activates apoptosis and autophagy pathways, leading to cell death. It elevates reactive oxygen species (ROS) levels, promoting apoptosis via increased cleaved caspase-3, while concurrently inhibiting the NF-kB signaling pathway by disrupting the interaction between p65 and FOXM1/β-catenin, both in vitro and in vivo [26,54]. Furthermore, niclosamide stimulates autophagy by activating the LC3B protein to induce cell death [56]. Niclosamide’s potential in non-small cell lung cancer (NSCLC) is evident as it effectively inhibits STAT3 phosphorylation and PD-L1 expression in a dose- and time-dependent manner [52]. Notably, it enhances the efficacy of PD-L1 antibodies and the PD-1/PD-L1 immune checkpoint blockade, thereby augmenting antitumor immunity [25]. In triple-negative and basal-like breast cancer cells, niclosamide not only impedes Wnt/β-catenin signaling but also intercepts ionizing radiation-induced Wnt/β-catenin signaling [31,33,53]. These effects arise from the downregulation of the Wnt ligand receptor, LRP6, and β-catenin. Moreover, in cisplatin-resistant cells, niclosamide or its combination with cisplatin restricts cell proliferation in vitro and in vivo through the Akt, ERK, and Src signaling pathways, while simultaneously suppressing the expression of snail and vimentin, effectively reversing the epithelial-to-mesenchymal transition (EMT) phenotype.

In our current study, niclosamide treatment of four DLBCL cell lines (U2932, HT, SU-DHL-5, and RC-K8) reduced cell proliferation/viability and G9a expression in a dose-dependent manner (Figure 2 and Figure 3). Notably, we have uncovered a novel aspect wherein niclosamide exhibits the ability to hinder DLBCL cell proliferation by downregulating the expression of the methyltransferase G9a. Concomitantly, there were increased levels of the autophagy signal gene LC3I/II compared to the untreated control, from G9a inhibited by niclosamide. Additionally, the binding interaction between niclosamide and G9a was investigated via molecular docking simulations. Utilizing the CB-DOCK2 web server, we explored potential binding sites and calculated Vina scores ranging from −7.9 to −7.2 for cavities with volumes between 1333 and 5316 Å^3^. The highest Vina score (−7.9) was observed in C3, which had a cavity volume of 2503 Å^3^ (Figure S3). The Vina score represents the binding energy, and a more negative score indicates a more stable binding interaction between the protein and ligand. Thus, the results suggest that niclosamide interferes with the activity and stability of G9a, which may have implications for its anticancer mechanism of action. Consistent with these reports, it was hypothesized that niclosamide can inhibit the proliferation of DLBCL by blocking G9a expression and inducing autophagy through LC3 expression. Furthermore, it was observed that autophagic flux was augmented from upstream to downstream by 5 μM niclosamide, as evidenced by the upregulation of autophagic-associated genes p62, BECN1, and LC3 (Figure 4). This suggests that niclosamide interferes with the activity and stability of G9a and involves autophagy-mediated cell death. Previously, niclosamide effectively inhibited the B lymphoma cell lines and T-cell leukemia through apoptosis and the oncogene protein Tax [57,58]. G9a regulates cancer progression through autophagy, a highly regulated catabolic process stimulated by various stressors [16,59,60]. G9a has been shown to suppress the autophagy signaling pathway and promote cancer cell growth [19]. Knockdown of G9a or treatment with G9a inhibitors significantly reduces cell proliferation by inducing cell cycle arrest and promoting autophagy, as evidenced by a decrease in H3K9 monomethylation levels [16]. Furthermore, it has been suggested that G9a directly binds to the promoters of LC3B, thereby facilitating the formation of autophagosomes, which is a critical step in autophagy [59]. The role of autophagy in cancer remains controversial, as it can promote tumor survival, while long-term and intense autophagy can lead to cancer cell death, known as autophagy-related cell death [61,62]. A commonly used method for analyzing autophagy is to study the accumulation of LC3B-II and to analyze the expression of its upstream genes.

Niclosamide, with its demonstrated potential to inhibit G9a expression and suppress DLBCL cell growth in this study, holds promise for future research and potential applications in various areas. Niclosamide could be explored in combination with other therapeutic agents, such as chemotherapy drugs or targeted therapies, to enhance its efficacy in treating DLBCL. Combinatorial approaches can help overcome drug resistance and improve patient outcomes. Further investigations are needed to elucidate the precise mechanisms by which niclosamide inhibits G9a expression and affects autophagy-related genes in DLBCL. Understanding these mechanisms can provide insights into the underlying biology of DLBCL and potentially uncover additional therapeutic targets.

This study has some limitations. First, it was a retrospective study with a limited number of DLBCL patients which limited the scope of our conclusion. Secondly, we lacked normal control specimens to compare the difference with DLBCL; therefore, we did not comprehensively analyze the variance of G9a expression in the DLBCL and normal control groups. Hence, it was only feasible to compare DLBCL patients at various cancer stages, and the categorization into two distinct groups for comparison purposes was contingent on the number of G9a-expressing cells. Thirdly, niclosamide suppresses DLBCL by reducing G9a expression and inducing autophagy in cellular experiments; thus, further research is necessary to confirm these results. However, the clinical samples and cellular experimentation have provided evidence of the correlation between G9a and DLBCL.

## 5. Conclusions

In light of the current knowledge, the precise interplay between G9a and niclosamide remains unexplored in the existing literature. Thus, further investigations are essential to elucidate the specific interactions and underlying mechanisms by which G9a and niclosamide may cooperate in the context of cancer development and therapeutic interventions. This study demonstrated high G9a RNA and protein expression based on a GEPIA and an IHC analysis. Niclosamide exhibits antiproliferative activity in DLBCL cell lines, decreases G9a expression, and induces production of p62 and BECN1, as well as LC3-dependent autophagy. Furthermore, niclosamide is a potent anticancer medicine that inhibits G9a and induces autophagy cellular pathways in DLBCL.

## Figures and Tables

**Table 1:** The primer sequences of RT-qPCR.

	Forward Primer	Reverse Primer
p62	5′-GTGAATTTCCTGAAGAACGTTGG-3′	5′-TGGAACTCTCTGGAGAGACGG-3′
BECN1	5′-CTGGACACGAGTTTCAAGATCCT-3′	5′- GTTAGTCTCTTCCTCCTGGGTCTCT-3′
LC3	5′-TCCTGGACAAGACCAAGTTTTTG-3′	5′-ACCATGCTGTGCTGGTTCAC-3′
GAPDH	5′-CTTAGCACCCCTGGCCAAG-3′	5′-ATGTTCTGGAGAGCCCCG-3′

**Table 2:** The clinical characteristics of DLBCL patients according to G9a expression.

	G9a > 40%	G9a < 40%	
	*N* = 21	*N* = 18	*p* Value
Age, (mean ± SD)	59.76 ± 18.86	61.22 ± 12.69	0.776
Gender, N (%)			0.878
Male	11 (52.38%)	7 (38.89%)	
Female	10 (47.62%)	11 (61.11%)	
ECOG, N (%)			0.717
1 + 2	20 (95.24%)	17 (100%)	
3 + 4	1 (4.76%)	1 (0.00%)	
Ann Arbor stage, N (%)			0.00166 **
I	0 (0%)	5 (27.78%)	
II	1 (4.76%)	7 (38.89%)	
III	7 (33.33%)	1 (5.55%)	
IV	13 (61.91%)	5 (27.78%)	
I-II	1 (4.76%)	12 (66.67%)	0.0158 *
III-IV	20 (95.24%)	6 (33.33%)	
BW (mean ± SD)	60.85 ± 9.27	61.50 ± 11.94	0.855
BH (mean ± SD)	162.94 ± 8.52	158.71 ± 6.38	0.105
BMI (mean ± SD)	23.02 ± 3.69	24.12 ± 4.01	0.397
WBC (mean ± SD)	6453.68 ± 2459.36	7365.88 ± 3487.83	0.367
Platelet (×10^3^, mean ± SD)	217.91 ± 105.5	259 ± 141.4	0.327
LDH (mean ± SD)	484.84 ± 474.77	255.59 ± 271.25	0.089
Beta-2 microglobulin (mean ± SD)	387.47 ± 347.70	404.75 ± 502.06	0.905
Albumin (mean ± SD)	3.89 ± 0.41	3.91 ± 0.64	0.922
GOT (mean ± SD)	32.63 ± 22.80	31.59 ± 12.00	0.867
GPT (mean ± SD)	24.11 ± 25.78	30.18 ± 18.30	0.426

* *p* < 0.05 and ** *p* < 0.01; ECOG, Eastern Cooperative Oncology Group; SD, standard deviation; WBC, white blood cell; LDH, lactate dehydrogenase; GOT, glutamic oxaloacetic transaminase; GPT, alanine aminotransferase.

## Data Availability

The data supporting the results of this study are available from the corresponding author upon reasonable request.

## Supplementary Materials

The following supporting information can be downloaded at https://www.mdpi.com/article/10.3390/cancers15164150/s1, Figure S1: The mRNA expression levels of G9a in difference cancers and normal patient tissues were analyze via GEPIA, Figure S2: The expression of G9a in different subtypes of DLBCL GCB and ABC, Figure S3: Molecular docking of niclosamide with G9a.
