# Supplementary material for: High G9a Expression in DLBCL and Its Inhibition by Niclosamide to Induce Autophagy as a Therapeutic Approach
Source: Cancers (Basel). 2023 Aug 17;15(16):4150. doi: 10.3390/cancers15164150 (PMC10452841; doi:10.3390/cancers15164150)
Supplement: Supplementary file 1 [file cancers-15-04150-s001.zip › cancers-2456520-supplementary.pdf]

Figure S1.

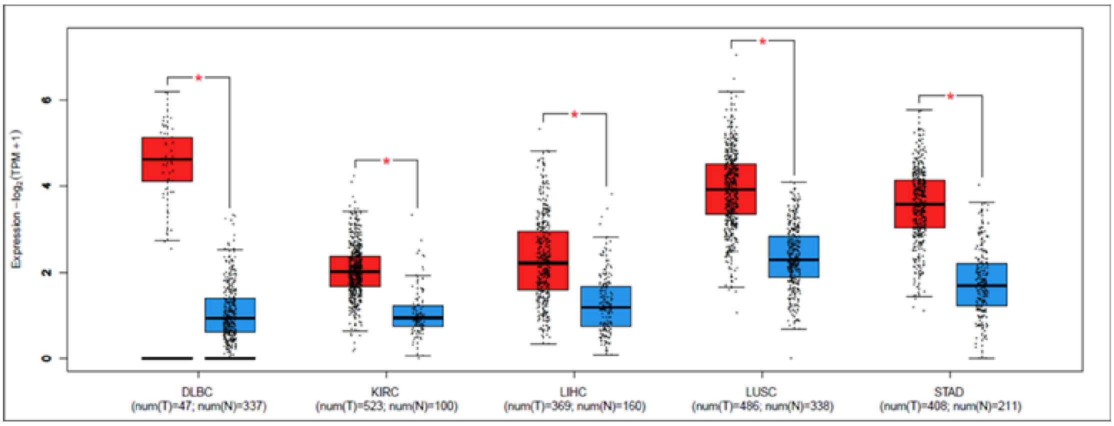

Figure S1. The mRNA expression levels of G9a in difference cancers and normal patient tissues were analyze via GEPIA. The red and blue boxes represent cancerous and normal tissues respectively. The y-axis represents log2(TPM+1) transformed RNA seq expression data. G9a mRNA expression levels were elevated in tumor tissues and significant differences were observed in those cancers. KIRC: kidney renal clear cell carcinoma, LIHC: liver hepatocellular carcinoma, LUSC: lung squamous cell carcinoma, STAD: stomach adenocarcinoma.

Figure S2.

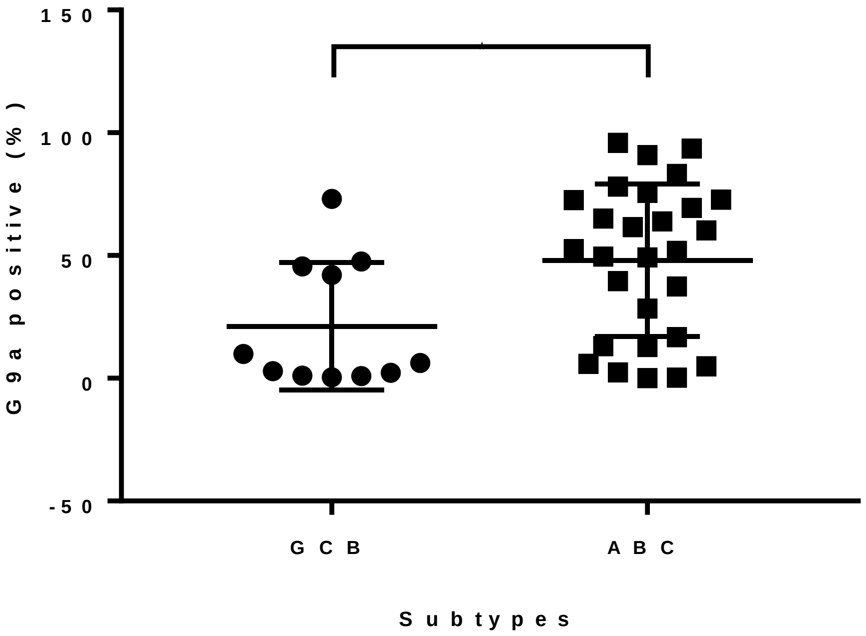

Figure S2. The expression of G9a in different subtypes of DLBCL GCB and ABC. Statistically significant difference between DLBCL GCB and ABC subtypes (*p<0.05)

Figure S3.

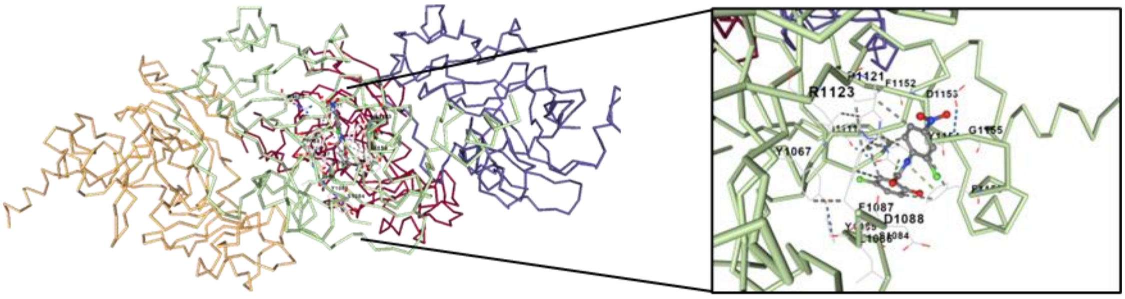

ID	Vina score	Cavity volumn (Å)	Center (X, Y, Z)	Docking size (X, Y, Z)
C3	-7.9	2503	32, 88, 85	22, 22, 22
C5	-7.8	1333	50, 104, 46	22, 22, 22
C2	-7.6	5237	33, 85, 42	22, 22, 22
C1	-7.5	5316	16, 68, 81	22, 22, 22
C4	-7.2	1512	27, 75, 63	22, 22, 22

Figure S3. Molecular docking of niclosamide with G9a.
